# AlN formation by an Al/GaN substitution reaction

**DOI:** 10.1038/s41598-020-69992-y

**Published:** 2020-08-03

**Authors:** Marsetio Noorprajuda, Makoto Ohtsuka, Masayoshi Adachi, Hiroyuki Fukuyama

**Affiliations:** Institute of Multidisciplinary Research for Advanced Materials (IMRAM), Tohoku University, 2-1-1, Katahira, Aoba-ku, Sendai, 980-8577 Japan

**Keywords:** Materials for devices, Materials science, Materials for optics

## Abstract

Aluminium nitride (AlN) is a promising semiconductor material for use as a substrate in high-power, high-frequency electronic and deep-ultraviolet optoelectronic devices. We study the feasibility of a novel AlN fabrication technique by using the Al/GaN substitution reaction method. The substitution method we propose here consists of an Al deposition process on a GaN substrate by a sputtering technique and heat treatment process. The substitution reaction (Al + GaN = AlN + Ga) is proceeded by heat treatment of the Al/GaN sample, which provides a low temperature, simple and easy process. *C*-axis-oriented AlN layers are formed at the Al/GaN interface after heat treatment of the Al/GaN samples at some conditions of 1473–1573 K for 0–3 h. A longer holding time leads to an increase in the thickness of the AlN layer. The growth rate of the AlN layer is controlled by the interdiffusion in the AlN layer.

## Introduction

Aluminium nitride (AlN) is a promising semiconductor material for use as a substrate in high-power, high-frequency electronic and optoelectronic devices. It can be used as a substrate in AlGaN-based ultraviolet C (UV-C) optoelectronic devices owing to its wide bandgap (above 6 eV)^1^, UV transparency^2^, and close lattice constant with that of AlGaN^3^. AlN can be grown in two forms: film and bulk. AlN films have been fabricated by various methods, such as metal–organic vapour-phase epitaxy (MOVPE)^4,5^, hydride vapour phase epitaxy (HVPE)^6,7^, pulsed laser deposition (PLD)^8,9^, molecular beam epitaxy (MBE)^10,11^, or sputtering^12,13^, to improve its crystalline quality, surface area, growth rate, or lower its processing temperature. Annealing techniques have been demonstrated to improve the crystalline quality of AlN films^14–16^.

To facilitate the further development of AlN crystal growth, several researchers have developed original and unconventional techniques. For example, the pyrolytic transportation method^17^, the liquid phase epitaxy (LPE) method using a Ga-Al binary solution^18^, Al-Sn flux growth^19^, AlN fabrication by using Al and Li_3_N solid sources^20^, and elementary-source vapour-phase epitaxy (EVPE)^21^ have been demonstrated. In the pyrolytic transportation method^17^, α-Al_2_O_3_ is used as an Al-source material, and it is heated at 2223 K to form Al_2_O gas in the nitrogen gas flow. The Al_2_O gas is transported to the growth zone to react with nitrogen gas at 2023 K on a sintered AlN plate for 30 h, which yields a rod-like AlN crystal (48-mm long). The advantages of this method are an economically friendly α-Al_2_O_3_ source and good crystalline quality of AlN. Wu et al*.*^21^ used metallic Al and nitrogen gas as source materials to grow an AlN crystal, which they called elementary-source vapour-phase epitaxy (EVPE). They grew the AlN with a growth rate of 18 μm/h under an optimum growth zone temperature of 1823 K. The advantages of this method are that it is conducted at a temperature lower than that of the sublimation method using no hazardous gas. Regarding the LPE methods, Adachi et al.^18^ grew a 1-µm-thick *c*-axis-oriented AlN layer on nitrided *c*-plane sapphire using a Ga-40 mol% Al flux with nitrogen gas injection at 1573 K for 5 h. The X-ray rocking curve full width at half maximum (XRC-FWHM) of AlN (0002) and AlN (10–12) were 50 and 590 arcsec, respectively. The advantages of this method are the uses of a moderate growth temperature and atmospheric pressure. Song et al*.*^19^ grew AlN single crystals with a size of 50 μm using an Al–Sn melt under a nitrogen gas atmosphere. Kangawa et al*.*^20^ also fabricated an AlN layer on an AlN seed by using Al and Li_3_N solid sources. Some of the above-mentioned methods might have the potential to grow bulk AlN crystals.

AlN in the bulk form is necessary to obtain the best performance of optical and electronic devices because of its significantly low threading dislocation density compared with AlN films grown on hetero-substrates. The AlN bulk crystal has been mostly grown through physical vapour transport (PVT)^22,23^ and HVPE^24,25^. The PVT technique is essentially the only method to fabricate high-quality crystalline AlN^26,27^. However, the PVT technique requires a high growth temperature of approximately 2473 K, which consumes a lot of energy and can be expensive. By lowering the growth temperature, a green AlN manufacturing process can be achieved with a reasonable cost. The HVPE technique is usually conducted at a temperature below that of the PVT. However, the HVPE technique requires high-quality PVT-AlN substrates to obtain a low threading dislocation density. Thus, it seems that no further developments can be made in the common AlN fabrication technologies.

To determine a novel technique for growing AlN to increase the possibility of further developments, here, we introduce an Al/GaN substitution reaction method. In this method, an Al layer deposited on a GaN substrate is used as a precursor, and AlN is obtained by the interfacial reaction between Al and GaN. The details are given in the next section. There are two purposes of this study. The first is to develop a novel technique to potentially grow a bulk AlN crystal by a substitution reaction of Al and GaN. The second is to fabricate an AlN film/GaN substrate structure that is also useful in some devices, for example, as a substrate in AlGaN/AlN/GaN high-electron-mobility transistors^28,29^ or as an insulated gate in AlN/GaN heterostructure field-effect transistors^30,31^. To the best of our knowledge, there have only been a few reports on fabricating AlN by heat treatment of Al on a GaN substrate. Luther et al. obtained a 2–3-nm-thick AlN layer by the heat treatment of Ti/Al and Pd/Al on GaN under Ar atmosphere at 600 °C for 15 and 30 h^32^. Moreover, Wang et al. demonstrated the advantage of Al buffer layers to obtain high-quality and stress-free GaN epitaxial films on Si substrates. In their study, an Al buffer layer with a thickness of 20–40 nm was grown on a Si substrate and, subsequently, a 250-nm-thick GaN film was grown on the Al buffer layer at 1123 K by PLD. The AlGaN peak was observed in the X-ray diffractometer (XRD) 2*θ − ω* scan profiles. However, AlN was not obtained^33^.

This paper focuses on the investigation of AlN fabrication by the substitution method at relatively low temperatures (around 1573 K). In this method, GaN was used as a starting material because it has the same wurtzite structure as AlN and has close lattice constants with those of AlN^34,35^. Currently, GaN is commercially available from several companies and institutions. There has been a tremendous amount of research on GaN. It has already been grown and investigated by the HVPE method^36,37^, ammonothermal method^38,39^, Na flux method^40–43^ and high-pressure solution growth method as reported in a review by Amano^44^. Although the GaN substrate is still expensive, large size GaN substrates will hopefully be available at a reasonable cost in the near future based on the ongoing intensive studies^45^. The substitution method we propose here consists of only Al deposition on a GaN substrate by a sputtering technique and heat treatment process, which provides some benefits such as much lower growth temperature, compared with the sublimation method (2473 K), and a simple and easy process. This study introduces the details of the Al/GaN substitution method including some fundamental results and discussion on the growth mechanism.

## Principle of Al/GaN substitution method

**Figure 1:**
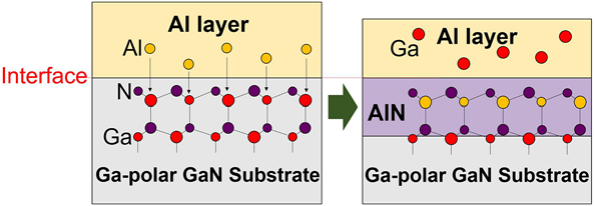
Schematic diagram of the Al/GaN substitution method.

Figure 1 shows a schematic diagram of the Al/GaN substitution method. This process starts with an Al layer deposited on a GaN bulk crystal, as shown in Fig. 1 (left). AlN is thermodynamically more stable than GaN. Ga atoms in the GaN can be substituted by Al atoms during heating the sample in an inert gas atmosphere. Thus, an AlN layer forms at the Al/GaN interface by the substitution reaction (1). This process proceeds with time by atomic diffusion through the AlN layer. The driving force of the reaction and mass transport can be controlled by selecting the temperature.

1 $${\text{Al}}\,\left( {\text{l}} \right) + {\text{GaN}}\,\left( {\text{s}} \right) \rightleftarrows {\text{AlN}}\,\left( {\text{s}} \right) + {\text{Ga}}\,\left( {\text{l}} \right)$$

From the thermodynamic point of view, the standard Gibbs energy of reaction (1) is determined by the following equation below:

2 $$\Delta G^\circ = \Delta_{{\text{f}}} G^\circ_{{{\text{AlN}}}} - \Delta_{{\text{f}}} G^\circ_{{{\text{GaN}}}}$$

where the standard Gibbs energies of formation of AlN and GaN, $\Delta_{{\text{f}}} G^\circ_{{{\text{AlN}}}} \,{\text{and}}\,\Delta_{{\text{f}}} G^\circ_{{{\text{GaN}}}}$, are − 134.2^46^ and 21.1 kJ/mol^47^, respectively, at 1573 K. Thus, the standard Gibbs energy of reaction (1) is − 155.3 kJ/mol, which implies the reaction spontaneously proceeds to the right-hand side.

## Results

### Thermal analysis

**Figure 2:**
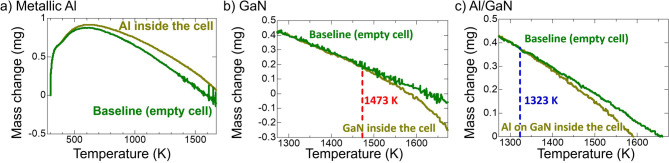
TG–DSC profiles of (**a**) metallic Al, (**b**) GaN and (**c**) Al/GaN.

Prior to the Al/GaN substitution reaction experiment, the thermal stabilities of metallic Al, single crystalline GaN and an Al layer deposited on GaN (Al/GaN) were studied by thermogravimetry–differential scanning calorimetry (TG–DSC). Figure 2 shows the TG–DSC profiles of these materials in an Ar atmosphere. Figure 2a shows that the profile of metallic Al was almost parallel to the profile of an empty cell (as a baseline). This implied that vaporization of Al is not significant up to 1673 K. However, the GaN profile started to exhibit a mass reduction from its baseline at 1473 K (as shown by the red dashed line in Fig. 2b). This implied that the GaN started to dissociate into Ga and nitrogen gas at 1473 K according the following reaction:

3 $${\text{GaN}}\left( {\text{s}} \right) \rightleftarrows {\text{Ga}}\left( {\text{l}} \right) + \frac{1}{2}{\text{N}}_{2} \left( {\text{g}} \right)$$

Meanwhile, The TG–DSC profile of Al/GaN started to show a mass reduction from its baseline at 1323 K (see the blue dashed line in Fig. 2c), 150 K lower than that for the GaN dissociation. If the substitution reaction (1) takes place alone, no mass reduction would occur. However, the GaN dissociation reaction (3) can take place together with reaction (1) at a lower temperature, because the Ga activity is greatly reduced by mixing Ga with Al.

### Al/GaN substitution reaction

#### Cross-sectional SEM image

**Figure 3:**
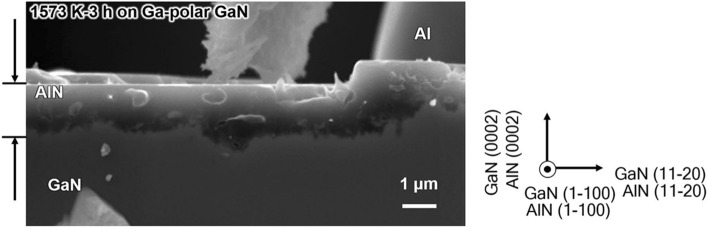
Cross-sectional SEM image of the AlN layers obtained after heat treatment of Al/GaN sample at 1573 K for 3 h.

Figure 3 shows the cross-sectional scanning electron microscopy (SEM) image of the AlN layer formed on the GaN substrate by the substitution reaction: A 7.6-μm-thick Al layer deposited on Ga-polar GaN substrate was annealed in an Ar atmosphere for 3 h at 1573 K. The AlN layer had the same crystal orientation with that of the GaN substrate, which will be explained by the in-plane crystallographic relationship described later.

#### Bird’s-eye view SEM–EDS images

**Figure 4:**
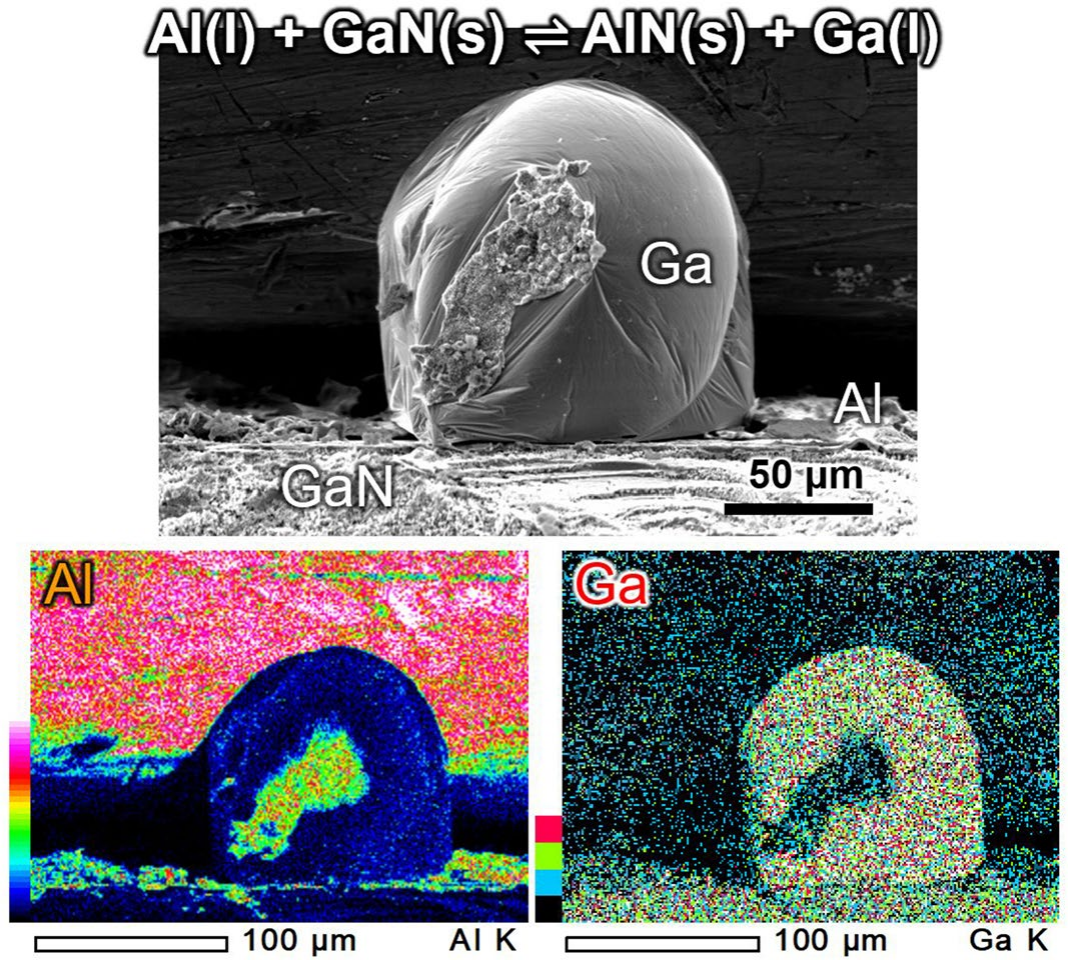
SEM-EDS images of a metallic Ga droplet observed above the Al layer after heat treatment of the Al/GaN sample at 1573 K for 3 h.

Figure 4 shows the bird’s-eye view SEM–EDS images of the Al on GaN substrate heat treated at 1573 K for 3 h. A metallic Ga droplet was observed on the Al/GaN sample. The Ga was formed by the substitution reaction (1) at the Al/GaN interface, and somehow it moved up to the surface of the Al/GaN sample. This is evidence of Ga formation by the substitution reaction. The AlN layer was hardly observed at this scale because the AlN thickness was only around 1.5 µm.

#### Crystalline orientation

**Figure 5:**
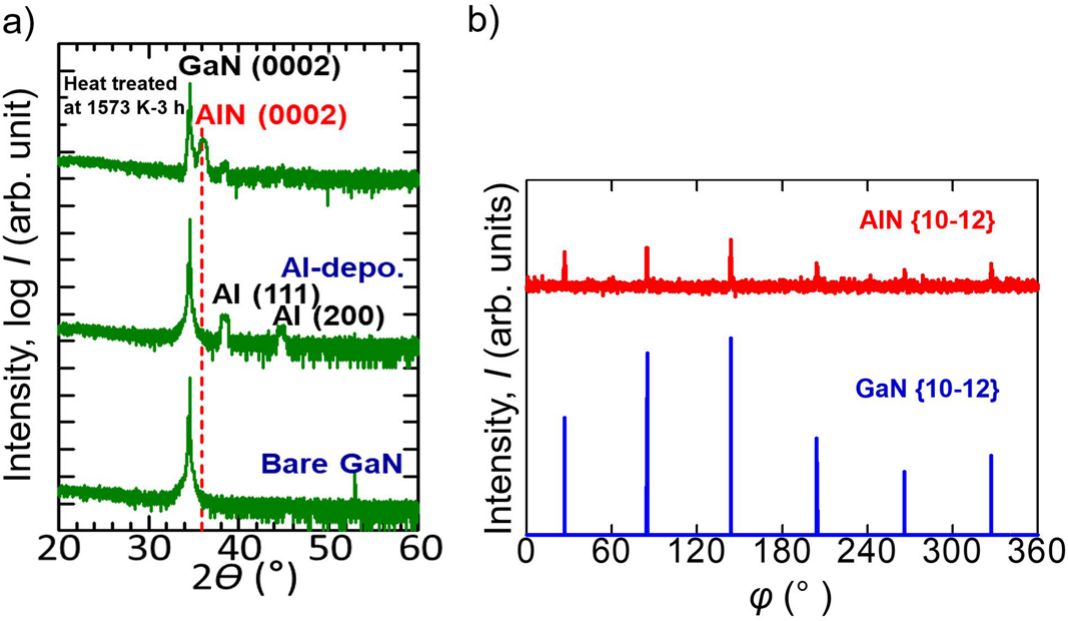
(**a**) XRD profile of the heat-treated Al/GaN sample at 1573 K for 3 h together with bare GaN and Al/GaN samples before heat treatment, (**b**) ϕ-scans of AlN {10–12} and GaN {10–12} for the Al/GaN sample after heat treatment at 1573 K for 3 h.

Figure 5a shows the XRD 2*θ − ω* scan profiles of the bare GaN substrate and Al/GaN samples with and without heat-treatment at 1573 K for 3 h. The profiles show that a *c*-axis-oriented AlN layer was obtained after heat treatment of the Al/GaN sample. The peak position of the AlN (0002) at a 2*θ* value of 36.0° reflections is also shown by the dashed line in Fig. 5a as a reference. AlN (0002) and GaN (0002) peaks were obtained for the heat-treated Al/GaN sample. Figure 5b shows the ϕ-scans of AlN {10–12} and GaN {10–12} for the heat-treated Al/GaN substrate. Both AlN {10–12} and GaN {10–12} exhibited 6 peaks, and they agreed with each other. From Fig. 5a,b, the in-plane crystallographic relationship between the AlN layer and GaN substrate is:

4 $${\text{AlN}}\,\left\{ {0002} \right\}//{\text{GaN}}\,\left\{ {0002} \right\}.$$

5 $${\text{AlN}}\,\left\{ {10{-}12} \right\}//{\text{GaN}}\,\left\{ {10{-}12} \right\}.$$

Ga peaks were not observed, which implied that the amount of formed Ga was too small to be detected by XRD. A certain amount of GaN may dissolve in AlN forming an Al_*x*_Ga_1−*x*_N solution, which would cause the blunt peak of AlN (0002). The formation of Al_*x*_Ga_1−*x*_N will be described in the cross-sectional transmission electron microscope (TEM) observation section.

#### Lattice constant

**Figure 6:**
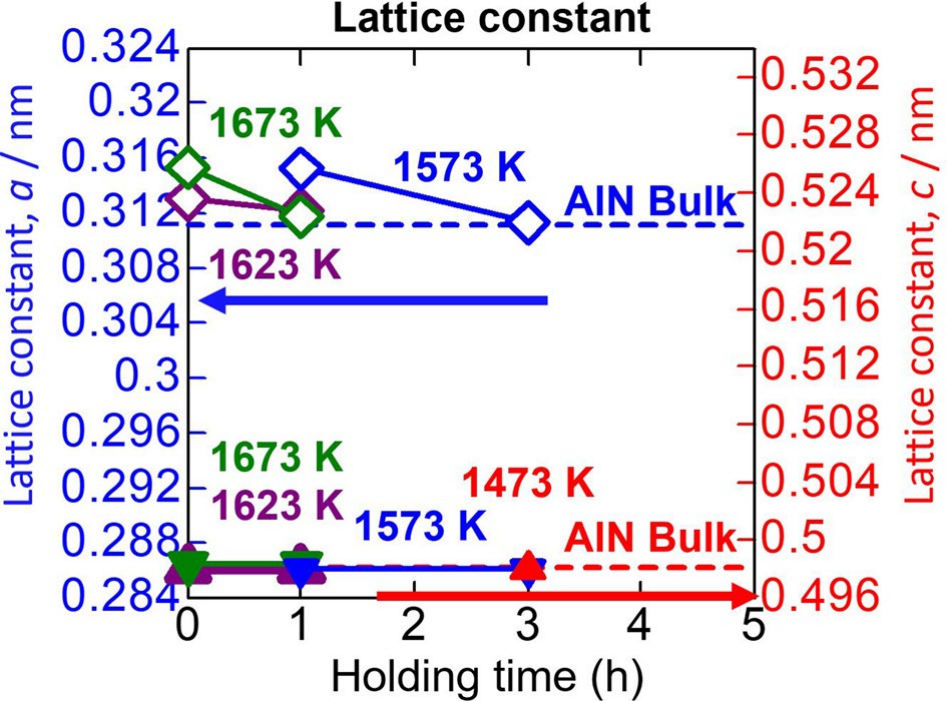
Lattice constants *a* and *c* of the AlN layers obtained after heat treatment of Al/GaN samples with various heat treatment temperatures and holding times together with those of bulk AlN.

Figure 6 shows the lattice constant of the AlN layers obtained after heat treatment of Al/GaN samples with various heat treatment temperatures and holding times. The lattice constants *c* of the AlN layers are almost the same with that of bulk AlN, but the lattice constants *a* are slightly larger than that of bulk AlN, and they approach that of bulk AlN with holding time.

#### Residual stress

**Figure 7:**
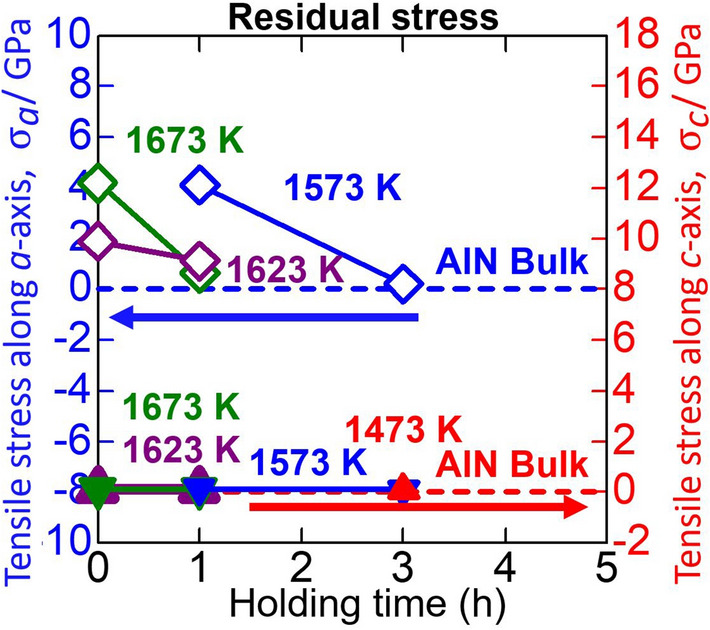
Residual stresses along the *a*- and *c*-axis of the AlN layers obtained after heat treatment of Al/GaN samples with various heat treatment temperatures and holding times together with those of bulk AlN.

Figure 7 shows the residual stresses of the AlN layers evaluated from the lattice constants presented in Fig. 6. The residual stresses along *c*-axis are almost zero. However, the residual tensile stresses along *a*-axes exist in the AlN layers and they approach zero with holding time. The thermal expansion coefficient along *a*-axis of GaN (6.2 × 10^–6^ K^−1^)^48^ is smaller than that of AlN (6.9 × 10^–6^ K^−1^)^49^, which may generate tensile stress along *a*-axis in the AlN layer near the AlN/GaN interface during cooling.

#### Crystalline quality of AlN

**Figure 8:**
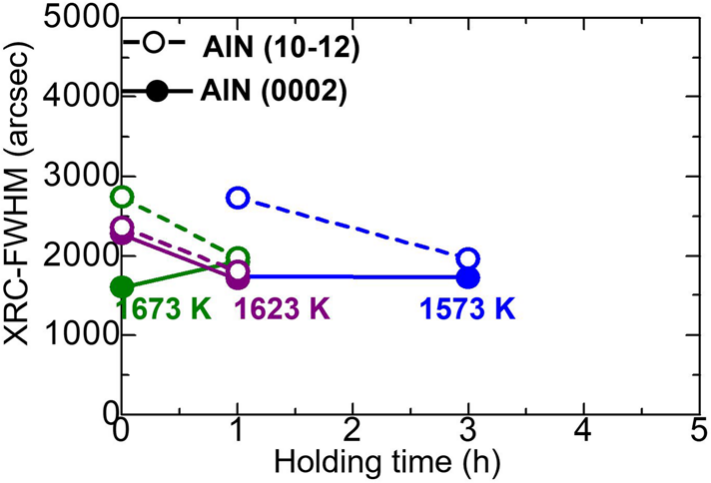
XRC-FWHM of AlN (0002) and AlN (10–12) after heat treatment of Al/GaN samples with various holding times of 0–3 h at temperatures of 1473–1673 K.

Figure 8 shows the XRC-FWHM of AlN (0002) and AlN (10–12) after heat treatment of Al/GaN samples with various holding times in the range of 0–3 h at temperatures of 1573–1673 K. Even though an AlN layer was obtained after heat treatment of Al/GaN at 1473 K for 3 h, its XRC-FWHM values are not shown owing to its low crystalline quality. The XRC-FWHM of AlN (10–12) decreased with increasing holding time. The XRC-FWHM values for GaN before heat treatment were in the range of 83–124 arcsec for GaN (0002) and 83–108 arcsec for GaN (10–12). Here, the XRC-FWHM values for the AlN obtained from the substitution method were quite large compared with the GaN substrate as a starting material. This could be because a certain amount of GaN non-uniformly dissolved in the AlN layer, as discussed in the next TEM observation section. The screw- and edge-type dislocations of the AlN layers were estimated from the XRC-FWHM^12^ at various heat treatment temperature and holding time, which are summarized in Table 1.

**Table 1:** Screw and edge dislocation densities in the AlN layers at various heat treatment temperatures and holding times together with those in the original GaN substrate estimated from XRC-FWHM (N/A means not available).

Heat treatment temperature (K)	Holding time (h)	Screw dislocation, *N*_S_/cm^−2^	Edge dislocation, *N*_E_/cm^−2^
1473	0	N/A	N/A
	1	N/A	N/A
	3	N/A	N/A
1573	0	N/A	N/A
	1	6.6 × 10^9^	2.5 × 10^10^
	3	6.5 × 10^9^	4.9 × 10^9^
1623	0	5.6 × 10^9^	2.8 × 10^10^
	1	8.1 × 10^9^	1.0 × 10^9^
1673	0	6.4 × 10^9^	1.9 × 10^9^
	1	8.0 × 10^9^	1.3 × 10^10^
*c*-plane GaN before heat treatment		1.5 × 10^7^	2.7 × 10^7^

#### Cross-sectional TEM observation

**Figure 9:**
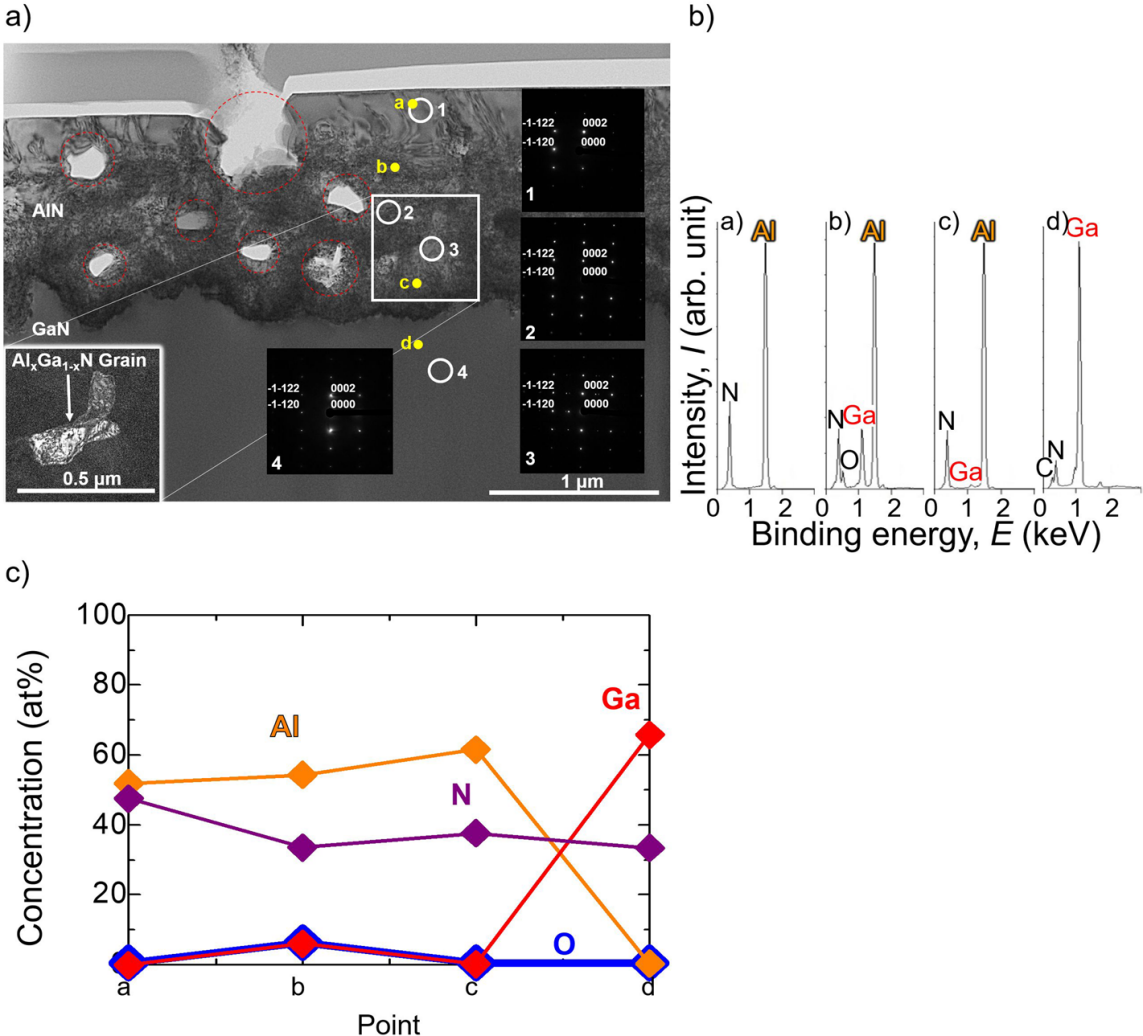
(**a**) Cross-sectional TEM image of the AlN layer obtained after heat treatment of Al on a GaN substrate at 1573 K for 3 h with an incident beam along GaN [1–100]. The Miller’s indices of AlN and GaN are also presented. (**b**) EDX spectra at points a, b, c of the AlN layer and point d of the GaN substrate shown in (**a**). (**c**) Concentrations of Al, Ga, N and O in at% at points a, b c of the AlN layer and point d of the GaN substrate shown in (**a**).

Figure 9a shows the cross-sectional TEM image of the AlN layer obtained after heat treatment of an Al/GaN sample at 1573 K for 3 h with an incident beam along GaN [1–100]. Thus, the AlN layer and GaN substrate could be clearly seen and distinguished from each other. It was observed that the AlN layer had a smooth surface, but the interface between AlN layer and GaN substrate was rough. In the AlN layer, some voids were observed (marked by the red-dashed-circles). The electron diffraction patterns of areas 1, 2, 3 and 4 (marked by the white circle) were measured. The Miller’s indices designated in areas 1, 2 and 3 belonged to the wurtzite structure of AlN (JCPDS file number 00–025-1133) and those in area 4 belonged to the wurtzite structure of GaN (JCPDS file number 00–002-1078). Areas 1 and 2 exhibited the same diffraction pattern as AlN; however, area 3 had some extra diffraction patterns in addition to the diffraction pattern of AlN. These extra diffraction patterns indicated the formation of a solid solution of Al_1−*x*_Ga_*x*_N in area 3 near the interface between AlN and GaN. To investigate this further, the AlN section marked by a white square was observed by dark-field-TEM, as shown at the bottom-left of Fig. 9a. It showed the grain consisted of Al, Ga and N, as indicated by the energy dispersive X-ray (EDX) spectra of point c shown in Fig. 9b. This several-hundred-nanometre sized Al_1−*x*_Ga_*x*_N grain was formed near the AlN/GaN interface, where Al diffused to GaN and partially substituted Ga at the Ga site forming Al_1−*x*_Ga_*x*_N before it completely formed AlN.

Figure 9b shows the EDX spectra at points a, b, c and d designated in Fig. 9a. Al and N peaks were detected at point a. However, Al and N peaks were detected in addition to a Ga peak at points b and c. Thus, Ga non-uniformly distributed in the AlN layer. The N peak intensities at points b, c, and d were lower than that at point a. This may imply that nitrogen atoms exited in the form of N_2_ gas, which resulted in some voids. Thus, the formation of N_2_ gas may cause the deviation of the TG curve of the Al/GaN sample from the baseline observed in Fig. 2c. The oxygen peak appearing at point b may have originated from contamination during the sputtering process of Al layer, and the oxygen was trapped in the AlN layer during the heat treatment. Figure 9c shows the concentrations of Al, Ga, N and O atoms in at% at points a, b, c and d. The AlN layer contains 6.3 at% O at point b, and 0.5–0.6 at% O at other points.

## Discussion

### Growth model of the substitution reaction method

**Figure 10:**
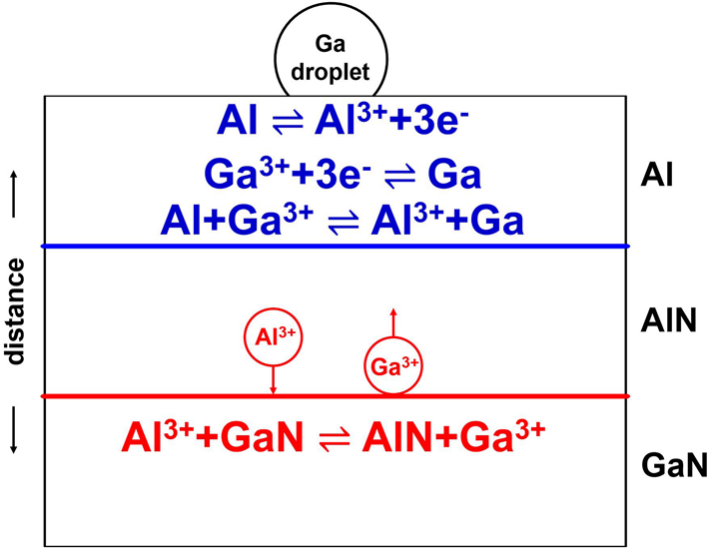
Growth model of AlN in the Al/AlN/GaN structure.

The growth model of the Al/GaN substitution reaction method is proposed as follows. Initially, Al reacts directly with GaN forming an AlN layer at the Al/GaN interface. A subsequent reaction occurs through the mass diffusion in the AlN layer. Figure 10 shows the growth model of an AlN layer in the Al/AlN/GaN structure. There are two interfaces: the Al/AlN and AlN/GaN interfaces. At the Al/AlN interface, metallic Al is oxidized to be Al^3+^, then it diffuses in the AlN layer towards the AlN/GaN interface. At the AlN/GaN interface, the Al^3+^ substitutionally reacts with GaN forming AlN and Ga^3+^. The Ga^3+^ then diffuses towards the Al/AlN interface, and it is reduced to be metallic Ga by the reaction with three electrons. The growth model is summarized as follows,

At the Al/AlN interface:

6 $${\text{Al}} \rightleftarrows {\text{Al}}^{3 + } + 3{\text{e}}^{ - }$$

7 $${\text{Ga}}^{3 + } + 3{\text{e}}^{ - } \rightleftarrows {\text{Ga}}$$

The total reaction at the Al/AlN interface is given by.

8 $${\text{Al}}\, + \,{\text{Ga}}^{{{3} + }} \, \rightleftarrows \,{\text{Al}}^{{{3} + }} \, + \,{\text{Ga}}$$

At the AlN/GaN interface:

9 $${\text{Al}}^{3 + } + {\text{GaN}} \rightleftarrows {\text{AlN}} + {\text{Ga}}^{3 + }$$

The overall reaction is given by the sum of reactions (6)–(9)

10 $${\text{Al}} + {\text{GaN}} \rightleftarrows {\text{AlN}} + {\text{Ga}}$$

The growth rate of AlN can be controlled by either interfacial reactions or interdiffusion. Assuming the interfacial reaction rates are much faster than interdiffusion, the growth rate is controlled by the diffusion.

### Kinetics of AlN growth

**Figure 11:**
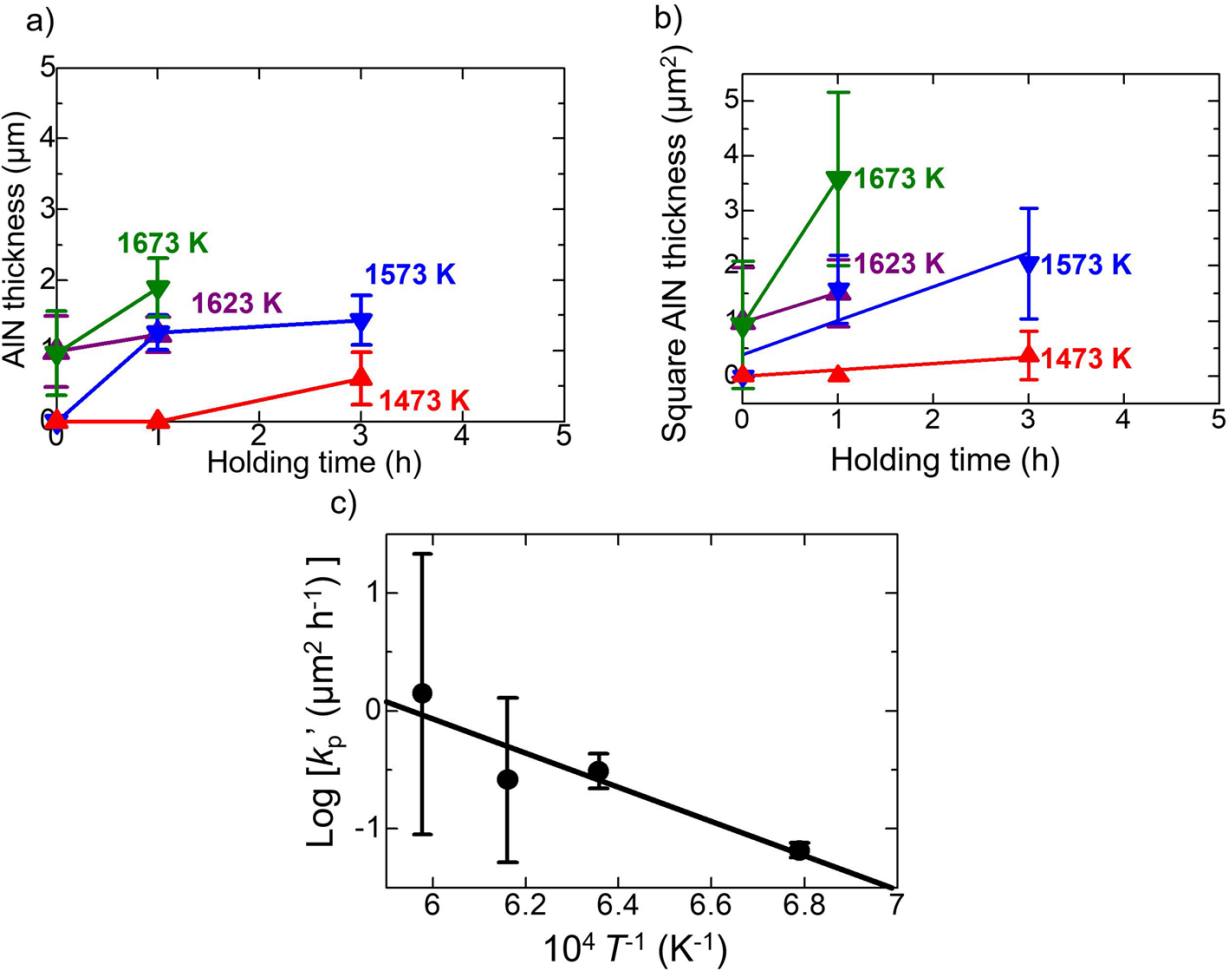
(**a**) Holding time dependence of the AlN thicknesses after heat treatment of the Al/GaN samples at 1473–1673 K. Error bars show the standard deviation of the AlN thickness. (**b**) Relation between square of the AlN thickness and holding time, and (**c**) Arrhenius plot of the AlN growth by the substitution reaction method.

Figure 11a shows the holding time dependence of the AlN thicknesses after heat treatment of the Al/GaN samples at 1473–1673 K. Here, the AlN thickness was measured from the cross-sectional SEM images.

There was no AlN layer formed at zero holding time at 1473 and 1573 K. This implied that the substitution reaction proceeds at a slow rate and needs time to form the AlN below 1673 K. The temperature effect on the thickness of AlN is difficult to observe because GaN decomposition is more aggressive in high temperatures (1623 and 1673 K) and affects the AlN thickness. The longer the holding time leads to the thicker AlN film. Assuming the parabolic rate law, Fig. 11a was revised as Fig. 11b. The parabolic rate constant *k*_*p*_*’* (μm^2^/h) is given by the following equation,

11 $$x^{2} = 2k_{p}^{\prime } t$$

here *x* (μm) is the AlN thickness and *t* (h) is the holding time. The Arrhenius plot is shown in Fig. 11c. The activation energy was calculated from the slope of the Arrhenius plot to be 121 ± 66 kJ/mol. The uncertainty is large owing to non-uniform AlN thickness after heat treatment of Al/GaN at temperatures of 1623 and 1673 K. This value has the same order with diffusion-controlled mechanism of some high-temperature oxidation studies of AlN. For instance, the activation energies for the oxidation of AlN obeying the parabolic rate law have been reported as 160 kJ/mol for the CaC_2_-doped AlN bulk at temperatures above 1523 K^50^ and 255 kJ/mol for the AlN bulk in the temperature range of 1173–1373 K^51^. The activation energies associated with the linear oxidation rate law of AlN have been reported as 175 kJ/mol for the AlN bulk in the temperature range of 1423–1623 K^52^ and 187 kJ/mol for the Y_2_O_3_-doped AlN bulk in the temperature range of 1323–1623 K^53^.

## Methods

### Sample preparation

Al films were deposited on Ga-polar GaN substrates using magnetron pulsed DC sputtering (Shimadzu, HSR552). An Al target (High Purity Chemicals, diameter: 101.6 mm, purity: 99.999 mass%) was used. A pulsed DC power of 600 W (Advanced Energy, Pinnacle Plus + 10 kW) was used with a frequency of the pulse of 100 kHz and a duty cycle of 60%. The square-wave pulse type was chosen. The distance between the target and the GaN substrate was 60 mm. The Ar gas (99.9999% purity) equipped with an oxygen filter (Nanochem Purifilter; Matheson PF-25 Serial number P02241) was introduced into the chamber with a flow rate of 1.7 × 10^−4^ L/s (10 sccm) and the total pressure was maintained at 0.6 Pa during sputtering. The oxygen filter removed NO_*x*_, SO_*x*_, H_2_S, < 0.1 ppb of H_2_O, O_2_, CO_2_, < 1 ppb of CO, and < 0.1 ppb of non-methane hydrocarbons from the argon gas. The growth temperature was fixed at 298 K. The sputtering time was 27 min that corresponded to 7.6-μm-thick Al on Ga-polar GaN substrates (Suzhou Nanowin Science and Technology Co. Ltd., size: 10 × 10.5 mm^2^, thickness: 350 ± 25 μm, crystal orientation: c-plane (0001), off-angle toward m-axis: 0.35° ± 0.15°, resistivity at 300 K: < 0.1Ω · cm, surface roughness of the front surface: Ra < 0.2 nm).

### Thermogravimetry–differential scanning calorimetry

The starting temperature for GaN dissociation was determined by a TG–DSC (Netzsch, STA 449 F3 Jupiter) measurement. The purge Ar gas with a flow rate of 50 mL/min and protective Ar gas with a flow rate of 20 mL/min at 1673 K were used. The total pressure was maintained at 0.1 MPa. The heating rate was kept at 1.67 × 10^−1^ K/s (10 K/min). Al wire standard material (Netzsch Gerätebau GmbH 99.999 mass%, diameter: 1.0 mm) and Al/GaN samples were heated to around 1673 K, then cooled to room temperature and then held there for 20 min. GaN sample was heated to 1673 K, cooled to 873 K and then kept at 873 K for 10 min. This procedure was repeated, and the sample was cooled to room temperature. The baselines using an empty cell were also measured.

### Substitution reaction experiment

**Figure 12:**
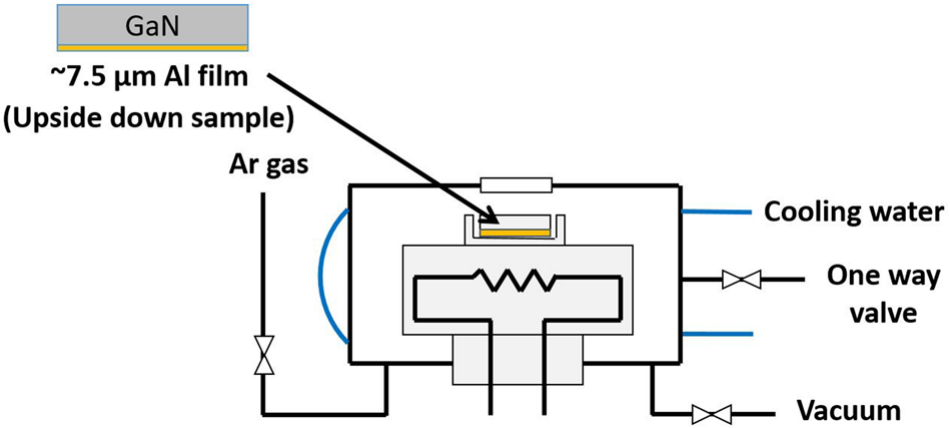
Schematic diagram of the heat treatment equipment.

Figure 12 shows a schematic diagram of the experimental setup for the Al/GaN substitution reaction experiment. The Al/GaN sample was placed upside-down. The samples were heated to the temperature range of 1473–1673 K in an Ar gas atmosphere with a flow rate of 30 mL/min at 293 K and cooled to room temperature after reaching each heat treatment temperature. From the TG–DSC result of Al/GaN in Fig. 2c, the starting temperature of Al/GaN substitution reaction was 1323 K. However, from Fig. 11, the AlN thickness at 1473 K even for 3 h was very small. Therefore, we selected 1473 K as the lowest experimental temperature. On the other hand, the GaN decomposition became more aggressive with increasing temperature as shown in Fig. 2b. Thus, the maximum temperature was selected at 1673 K, but it was applied only for the short-duration experiments less than 1 h. The heating and cooling rates were held constant at 10 K/min. The total pressure was kept at 106.5 kPa in the chamber. The holding time was varied from 0 to 3 h for 1473 and 1573 K, but only 0 and 1 h for 1623 and 1673 K.

### Sample characterization

The thickness, crystalline quality and cross-sectional image of the AlN layers formed between the Al layer and GaN substrate were evaluated around the middle part of the substrates**.** The interface morphology and the bird’s-eye view of the AlN layers were examined using a SEM (JEOL JCM-5700). The AlN thickness was evaluated from these images. The 2*θ − ω* scan profile, where 2*θ* is the diffraction angle between the incident X-ray and the detector, and *ω* is the incident angle between the incident X-ray beam and the sample surface, and the X-ray rocking curve (XRC) profile were obtained using an XRD (Bruker, D8 Discover MR). An X-ray source of Cu-Kα radiation was selected. The XRD system was equipped with two Ge (400) crystals in its monochromator and a single-bounce Ge (220) in the analyser. The voltage and current in the X-ray cylinder during the XRD measurement were 40 kV and 40 mA. The 2*θ − ω* scan was conducted with a step size of 0.05°. The ϕ-scan was performed with a 0.1° step size where ϕ is a rotational axis normal to the sample surface.

A TEM (Hitachi High Technologies, H-9000NAR), with an acceleration voltage of 200 kV and a magnification accuracy of ± 10%, was used to acquire the TEM images and electron diffraction patterns. An EDX equipped to the TEM system (Hitachi High-Technologies HD-2700) was used to carry out the elemental analysis at some points of the sample. The beam diameter was approximately 0.2 nm. Before the sample was measured by TEM and EDX, the remaining Al on the AlN was removed by wet etching using a 0.1 mol/L HCl aqueous solution at 353 K for 3 h, and then, the sample was pre-treated with a thinning process by focused ion beam (FIB) apparatus using the μ-sampling method.
